# Implementing Team-Based Post-Stroke Telerehabilitation: A Case Example

**DOI:** 10.5195/ijt.2022.6438

**Published:** 2022-06-03

**Authors:** Melissa Anderson, Brad Dexter, Ana Hancock, Nealey Hoffman, Steve Kerschke, Karen Hux, Dipika Aggarwal

**Affiliations:** 1 Kintinu Telerehab, Quality Living, Inc., Omaha, Nebraska, USA; 2 Department of Neurology, University of Kansas Health System, Kansas City, Kansas, USA

**Keywords:** Interdisciplinary teams, Service delivery models, Stroke treatment, Telehealth, Telerehabilitation

## Abstract

Access to extensive, interdisciplinary rehabilitation following stroke is necessary to optimize recovery. Telerehabilitation is an appropriate model for delivering these services. However, given its relatively recent increase in popularity as a service delivery model, researchers have yet to explore the feasibility of interprofessional coordination and collaboration as a guiding framework for telerehabilitation and the effects of team-based remote service delivery on recovery of body functions and activities. This case example reports the development, implementation, and progression of a post-acute treatment program delivered via telerehabilitation to a woman with left hemorrhagic stroke. As is typical, therapy time alone afforded insufficient practice to exploit neuroplasticity and ensure maintenance and generalization of improved functioning; hence, the team worked collaboratively to encourage interdisciplinary activities outside scheduled treatment sessions. Standardized and informal assessments administered at the start and conclusion of treatment confirmed improved functioning as did the client's progress toward independent living and return to work. Implications for telerehabilitation practices are discussed.

Timely access to extensive rehabilitation is essential for promoting neuroplasticity and optimizing functional recovery after stroke (Belagaje, 2017). As the coronavirus disease 2019 (COVID-19) pandemic persisted in 2020 and 2021, hospital systems had to postpone or cancel many in-person, post-acute rehabilitation services, thus substantially interfering with the application of best practices to post-stroke management (Buheji & Hassani, 2020; Stein et al., 2020). In response, telerehabilitation grew as an alternative delivery method for physical therapy (PT), occupational therapy (OT), and speech-language pathology (SLP) services (American Physical Therapy Association, 2020; Buheji & Hassani, 2020; Moradi et al., 2021; Prabawa et al., 2021; Stein et al., 2020; Tenforde et al., 2020). However, given that remote service delivery has only recently surged in popularity, researchers have yet to explore how reliance on accepted methods of best practice for stroke rehabilitation—such as incorporating a team-based approach to service delivery that incorporates interprofessional care plans and interdisciplinary team meetings (Careau et al., 2008; Clarke & Forster, 2015; Heuer et al., 2019; Stroke Unit Trialists' Collaboration, 2013; Yagura et al., 2005)—occurs with telerehabilitation. In particular, interdisciplinary coordination of therapeutic activities to implement team-based post-stroke telerehabilitation warrants examination as does the overall efficacy of the service delivery model for achieving clients' long-term goals.

Telerehabilitation can be an effective service delivery method for specific aspects of post-stroke care (Macoir et al., 2017; Tchero et al., 2018). For example, Cramer et al. (2019) compared upper extremity improvements for 124 adults with stroke receiving either telehealth or dose- and intensity-matched in-clinic treatment and found comparable improvement across groups. By monitoring exercise program participation by Veterans with chronic stroke receiving PT via telehealth, Miller et al. (2014) documented increases in the variety of exercises and number of repetitions performed. Regarding SLP, Meltzer et al. (2017) delivered either in-clinic or telepractice sessions to 44 adults with aphasia or cognitive-communication disorder secondary to stroke and, apart from communication confidence, found statistically equivalent pre- to post-intervention gains regardless of treatment delivery method.

Documentation of telerehabilitation's usefulness is important from the standpoint of establishing efficacy for individual aspects of post-stroke treatment; however, most people sustaining stroke experience deficits across a range of physical and communicative functions and receive concurrent treatment from an interdisciplinary team of professionals working collaboratively. Indeed, considerable evidence supports the notion that interdisciplinary, team-based approaches to stroke rehabilitation are superior to isolated treatments (Clarke & Forster, 2015; Stroke Unit Trialists' Collaboration, 2013; Yagura et al., 2005). Such approaches optimize rehabilitation by encouraging professionals to be creative in working collaboratively to meet unique and personalized client goals. Team-based approaches promote the sharing of expertise and professional skills across disciplines to recognize overlapping and supportive treatment opportunities that facilitate functional gains in domains important to a client (Clarke & Forster, 2015; Stroke Unit Trialists' Collaboration, 2013).

Recent restrictions to in-person treatment and increased reliance on telerehabilitation secondary to the COVID-19 pandemic give rise to questions about team-based procedures professionals can implement to provide mutually supportive intervention services. The purpose of this case example was to document the development, implementation, and progression of a person's post-acute treatment when delivered by an interdisciplinary team of telerehabilitation specialists. The case serves as an example of PT, OT, and SLP professionals working collaboratively with a client to achieve meaningful progress toward long term goals by incorporating functional therapeutic tasks in a natural environment that provides multiple opportunities for repetitive practice. We received permission to access case information from the Institutional Review Board before initiating any review of treatment records, and the subject of the case example, Dipika Aggarwal^1^, signed a consent form acknowledging her willingness to participate.

## CASE DESCRIPTION

**Figure 1:**
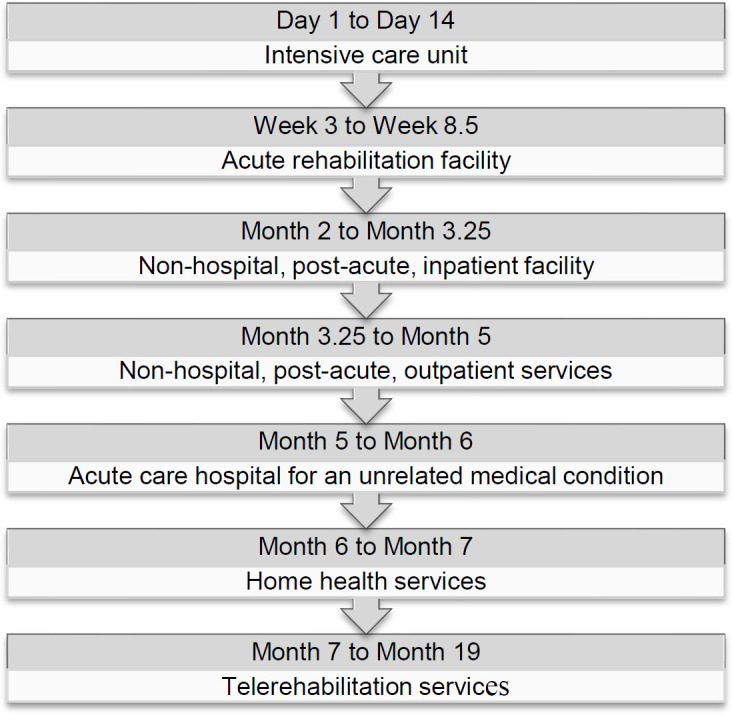
Progression of Medical and Rehabilitation Service Provision

Dipika was 37 years old, living independently, and working fulltime as a hospital-based neurologist when she sustained a moderate stroke (NIH Stroke Scale = 11). The etiology was a subarachnoid hemorrhage caused by a ruptured aneurysm at the left middle cerebral artery bifurcation. After neurosurgery and 2 weeks of medical stabilization in an intensive care unit, Dipika transferred to an acute rehabilitation facility for 5.5 weeks of treatment for right hemiplegia and aphasia. She then transferred to a non-hospital, post-acute, inpatient rehabilitation program for an additional 5 weeks of intensive PT, OT, SLP, life path, and counseling services followed by 2 months of daily outpatient PT, OT, and SLP services. An unrelated medical condition requiring surgical intervention then forced return to an acute care hospital. Recovery extended over several weeks, and, when ready to resume therapy for stroke-related deficits, COVID-19 restrictions limited Dipika's treatment options. For the next month, she received 30 minute PT and OT sessions three times per week and a total of two SLP sessions from a home health service provider. Dissatisfaction with progress prompted her to seek services via telerehabilitation as an alternative. She began telerehabilitation services 7 months post-stroke. Initial sessions in each discipline lasted 60 minutes and occurred three times weekly; the frequency and intensity of PT and OT sessions remained unchanged over the next year, whereas SLP sessions gradually declined to 30 minutes every other week. Figure 1 depicts the progression of Dipika's treatment through various medical and rehabilitation facilities and service providers.

Dipika's telerehabilitation team had previously worked on collaborative interdisciplinary teams within an inpatient rehabilitation setting. This experience provided them with substantial training and experience implementing team-based services, and they transferred many skills from their inpatient practice to telerehabilitation operations. However, because limited literature exists about applying an interprofessional approach to remote service delivery, the team engaged in ongoing learning and discussion to refine their communication strategies, meeting formats, and overall efficiency.

The first step pursued by Dipika's clinicians was to perform discipline-specific assessments at the initiation of telerehabilitation services. This served as the basis for developing a “problem list” and establishing preliminary functional goals. Dipika's reduced range of motion, strength, and fine motor coordination in the right upper extremity interfered with performing basic and instrumental activities of daily living; she relied primarily on her nondominant, left arm and hand to perform grooming, dressing, and feeding tasks, and she was dependent on family members for meal preparation and home management. Dipika's decreased stamina, balance problems, and gait abnormalities interfered with walking, and she used a single-point cane for stabilization. Regarding communication, Dipika's struggles with word retrieval limited conversational participation. She also needed support to comprehend and retain lengthy information presented through the auditory or written modality.

The clinical team held a preliminary meeting to share their assessment findings. As a clinician likely to have substantial involvement in Dipika's program, her physical therapist assumed primary ownership of the case and facilitated the 10- to 15-minute meeting to establish a comprehensive treatment plan and identify shared goals. In accordance with suggestions from TeamSTEPPS^®^ 2.0 (Agency for Healthcare Research and Quality, 2019), the structured meeting format encouraged an efficient yet thorough means of sharing of information and developing a treatment plan. The team leader then recorded details about the developed program in an easy-to-access, shared document that served as a guide for future meetings. Specifically, they modified the “I Pass the Baton” tool from TeamSTEPPS^®^ to one they called, “Pass It On.” Table 1 provides a “Pass It On” template with an explanation of type of content for inclusion in each category and Dipika's information inserted in the right hand column.

**Table 1:** Pass It on Content Categories, Explanations, and Case Example Information

Category		Explanation	Case example information
P	Patient	Patient demographics. Who is the patient as a person (i.e., interests, motivators)? Where does the person live? Diagnosis and details of injury?	Interests and motivators: Neurologist, devoted fiancée, caring family member, outdoor adventurer/world traveler, cardio exerciser. Home location: Currently living in California with brother but will eventually move to Missouri to live independently. Injury details: Began having blurry vision and confusion while at work. Head CT showed left subarachnoid hemorrhage and left middle cerebral artery bifurcation aneurysm.
A	Assessment	Primary concerns (i.e., problem list) for each discipline.	PT: Impaired strength, balance, endurance, and functional mobility. OT: Impaired strength, coordination, and functional use of right upper extremity. SLP: Difficulty with word finding, verbal expression, and reading/auditory comprehension.
S	Situation	Support system, caregivers, case manager, home environment, community resources/access, medical concerns/comorbidities.	Support system: Several family members including her mother, father, fiancé, brother, and sister. Caregivers: Father and caregiver will be present during therapy sessions. Home environment: Home in Missouri is not currently accessible—on the second floor without elevat or access. Medical comorbidity: History of other complex medical issues.
S	Safety concerns	Physical, cognitive, emotional.	Physical: Deconditioned and high risk for falls. Cognitive: Inability to express self-care needs fully and over-estimates abilities. Emotional: Mild, situational depression.
IT	IT setup/concerns	Connectivity, location, device(s) available/needed, understanding.	Connectivity: Will use a laptop and smartphone for virtual therapy sessions. No concerns with internet or cellular connectivity in the home or community.
O	Ownership	Clinical lead? Main contact for patient?	Clinical ownership: Physical therapist. Family ownership: Father and brother.
N	Next	Clarify treatment goals and plan of care. What will happen next? Frequency/duration of sessions? Anticipated treatment duration?	PT: 3x/week × 30 minutes per session. Goals: Recumbent bike for 15+ minutes, independent completion of home exercise program, ambulation outdoors on level surfaces at a distance and speed to allow for safe community access. OT: 3x/week × 30 minutes per session. Goals: Tolerate upper extremity weight bearing × 8 minutes, caregiver(s) demonstrate successful completion of a home exercise program, tolerate electrical stimulation for neuro re-education × 20 minutes daily. SLP: 3x/week × 60 minutes per session. Goals: Develop and carry-out a structured daily schedule, utilize a memory compensation tool, implement strategies for word-finding and social conversations, consistent recall of details from reading/auditory comprehension tasks. All disciplines will re-assess in 4-6 weeks to determine ongoing needs and adjust treatment plan as needed.

**Figure 2:**
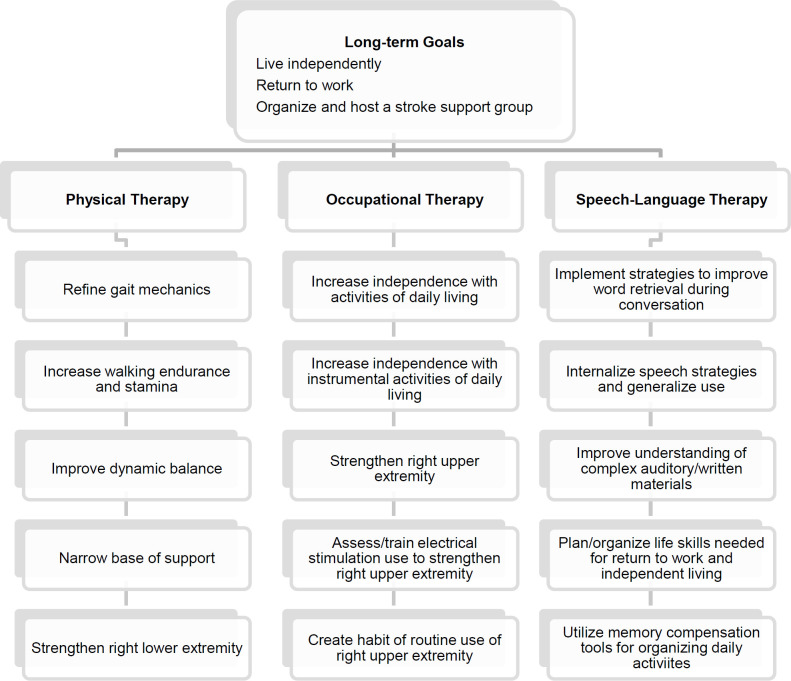
Discipline-specific Objectives to Facilitate Long-term Goal Achievement

The clinical team then met with Dipika to discuss her long-term goals. These included resuming independent living, returning to work, and organizing and hosting a stroke support group. The discipline-specific objectives appearing in Figure 2 served as a means of facilitating achievement of the long-term goals. Scheduled treatment sessions provided opportunities to present instructions and monitor exercise performance and strategy implementation. However, therapy sessions alone afforded insufficient practice to promote neuroplasticity and ensure maintenance and generalization of improved functioning. To address this problem, the treatment team met remotely every two weeks to brainstorm interdisciplinary ways of capitalizing on Dipika's intrinsic motivation, access to materials in her environment, and professional understanding of neuroplasticity concepts to implement therapy-related activities outside scheduled telerehabilitation sessions.

As the treatment team leader, Dipika's physical therapist assumed responsibility for keeping the program on track, facilitating communication with all stakeholders, and guiding other programmatic related discussions. He did this during five- to 10-minute check-ins that were part of a larger meeting at which the team reviewed all current and newly scheduled clients. Additionally, the team utilized Microsoft 365^®^ applications to support communication, collaboration, information sharing, and accountability; they used Microsoft Teams for instant chat and video calls and the Planner application to assign tasks and offer discussion topics for the bi-weekly meetings. Examples of interdisciplinary activities appropriate for Dipika and the disciplines targeted appear in Table 2; these example activities were supplemental to continued engagement in discipline-specific tasks.

**Table 2:** Interdisciplinary Activities to Augment Telerehabilitation Sessions

Physical and Occupational Therapy
Standing during personal hygiene and home maintenance tasks to increase balance and endurance Folding laundry in standing position while using electrical stimulation to right arm Using both hands to wash hair, wash dishes, or prepare food while in standing position Maneuvering through home environment while manipulating home maintenance equipment with right hand
Occupational and Speech-language Therapy
Planning and sequencing daily schedule and performance of home management tasks Implementing reading comprehension, memory, and word retrieval strategies while planning meals and shopping for food Engaging in conversation while performing activities of daily living Manipulating utensils with right hand while performing sequenced cooking tasks Manipulating medical instruments with right hand while practicing sequenced physical exams of patients
Physical Therapy and Speech-language Therapy
Treadmill training to refine gait mechanics and increase speed while processing auditory content Walking, stair climbing, and navigating uneven terrain while relaying summarized information to another person
Physical, Occupational, and Speech-language Therapy
Planning, sequencing, and following structured schedule for work, lesiure, and sleep routines Planning, sequencing, and performing work and leisure tasks using ergonomic set-ups and compensatory communication strategies

Dipika improved steadily over the one-year course of telerehabilitation services while residing with a family member. Results of standardized and informal assessments administered at the start and conclusion of telerehabilitation appear in Table 3 and provide evidence of improvement. Further evidence was Dipika's resumption of functional activities performed prior to her stroke and consistent with her desire to live independently and return to her previous employment. For example, at 9 months post-stroke, Dipika began assuming responsibility for preparing simple breakfast and lunch meals for herself; one month after that, she returned to performing home management tasks such as doing laundry; and, after another two months, she initiated resumption of work responsibilities by passing continuing education exams and observing neurosurgeries. By the conclusion of telerehabilitation services, Dipika was working remotely two days per week, coordinating and hosting a support group for people with stroke, and preparing to return to independent living.

**Table 3:** Standardized and Informal Assessment Results at the Initiation and Conclusion of Telerehabilitation

Assessment target	Performance at initiation of telerehabilitation	Performance at conclusion of telerehabilitation
Right upper extremity	Decreased active range of motion: 10% of total function Flexor synergy pattern involving bicep Wearing shoulder brace for transfers and functional mobility 1-finger shoulder subluxation Gross finger flexion of all digits; no finger extension Strength measures: biceps 3/5; triceps 0/5; wrist extension 0/5; wrist flexion 3/5 Minimal to moderate assistance for upper body dressing Modified assistance for lower body dressing Modified assistance for donning/doffing socks and shoes Total assistance for grooming hair (i.e., ponytail) Moderate assistance for showering while seated on tub transfer bench Modified independence for toileting Total assistance for simple and complex meal preparation Total assistance for home management tasks	Decreased active range of motion: 60% of total function Flexor synergy pattern involving bicep; improved to 90° of shoulder flexion without flexor synergy pattern Wearing shoulder brace for support while jogging; using kinesiology tape for shoulder support during daily activities ½-finger shoulder subluxation 10° of active flexion and 10° of active extension of proximal inter-phalangeal joints of 4^th^ and 5^th^ digits Strength measures: biceps 4/5; triceps 4-/5; wrist extension 3/5; wrist flexion 3/5; with elbow stabilized on table, 5° of wrist flexion Modified independence for upper body dressing Modified independence for lower body dressing Modified independence for donning/doffing socks and shoes Total assistance for grooming hair (i.e., ponytail) Modified independence for showering while standing Modified independence for toileting Modified independence for simple and complex meal preparation Modified independence for home management tasks
Right lower extremity	Normal range of motion Stiffness in calf and hamstring muscles that limit active movement Standing on left leg for 30 seconds; unable to stand on right leg Tandem standing: Not tested Requires 3 to 4 steps to turn around Requires extra time to move from sitting to lying position and vice versa Needs use of arms to transfer from sit to stand position from surface height lower than 18 in. Ambulating 0.4 – 0.5 miles outside with single point cane Treadmill gait speed while jogging: Unable to perform No arm swing while walking Step-over-step pattern with handrail when ascending stairs; step- to pattern with handrail when descending stairs Unable to transfer to and from floor Berg Balance Assessment: 46/56 Functional Gait Assessment: Not tested Activity-specific Balance Confidence Scale: Not tested	Normal range of motion Reduced strength in terminal knee extension, active dorsiflexion/eversion, and gluteus medius Standing on left leg for 60 seconds; standing on right leg for 60 seconds given cueing and contact guard assist Tandem standing for 1 minute with different foot positions Pivots and turns around quickly with no extra steps Moves from sitting to lying position and vice versa independently and within normal time limit Does not need use of arms to go from sit to stand position from surface height lower than 18 in. Ambulating up to 3 miles outdoors with no assistive device; uses an electrical orthosis for foot drop assistance Treadmill gait speed up to 3.7 mph for short stints while jogging Reduced arm swing while walking; needs cueing for heel strike on right side Step-over-step pattern without handrail when ascending stairs; step-over-step pattern with handrail when descending stairs Independently transfers to and from floor without stable object Berg Balance Assessment: 56/56 Functional Gait Assessment: 28/30 Activity-specific Balance Confidence Scale: 88%
Communication	Recalls 2/7 details after silent reading of single paragraph (Quicksand passage) Use of verbal summarization to demonstrate understanding and recall of content after silent reading of lengthy material: Not tested Recalls 2/6 details after listening to paragraph-length content (Gold Rush passage) Use of verbal summarization to demonstrate understanding and recall of content presented during 30-minutes interaction: Not tested Generates exemplars of common categories given 30 seconds: 7 fruits, 6 weather conditions, 3 words beginning with *m* Generates exemplars relating to professional terminology given 30 seconds: Not tested Self-report of attention and processing problems when listening Independently performs 6/6 steps for using calendar app on phone to schedule/structure day	Recalls 6/7 details after silent reading of single paragraph (Quicksand passage) Independent use of verbal summarization to demonstrate full understanding and recall of content after silent reading of lengthy material Recalls 4/6 details after listening to paragraph-length content (Gold Rush passage) Independent use of verbal summarization to demonstrate understanding and recall of content presented during 30-minutes interaction Generates exemplars of common categories given 30 s: 12 fruits, 7 weather conditions, 8 words beginning with *m* Generates exemplars relating to professional terminology given 30 s: 12 exemplars No self-report of attention and processing problems when listening Independently performs 6/6 steps for using calendar app on phone to schedule/structure day

## DISCUSSION

Growing evidence supports the appropriateness of relying on telerehabilitation for delivering services to people needing intensive treatment over multiple months (Buheji & Hassani, 2020; Cramer et al., 2019). Telerehabilitation services for the presented case started seven months post-stroke. Despite the length of time that had elapsed since stroke onset, the client's improvement was substantial and represented important progress toward independent living and work resumption goals. The improvement achieved provides evidence of the effectiveness of telerehabilitation for promoting recovery by people with chronic stroke challenges as well as the value of combining discipline-specific and interdisciplinary activities into a comprehensive treatment program.

Much extant research documenting the benefits of team-based approaches to stroke care relate to acute hospital or inpatient rehabilitation settings (e.g., Stroke Unit Trialists' Collaboration, 2017; Yagura et al., 2003, 2005); less information is available about the benefits of interdisciplinary teams on post-acute recovery. However, encouraging professionals to work in an interdisciplinary fashion is likely to increase efficacy regardless of recovery stage because of the reduction in service fragmentation and duplication that results from engagement in team planning. In addition, team-based approaches promote client-centered services that address individualized goals and enhance quality of life (Clarke, 2010; Markle-Reid et al., 2011).

Applying an interdisciplinary team approach to telerehabilitation has a similar potential to bolster treatment efficacy as it does for in-person service delivery. A critical factor, however, is that team efforts must extend beyond being merely multidisciplinary; only when team members share comparable status, exhibit mutual respect, and collaborate fully in an interdisciplinary fashion to make and implement treatment decisions are the benefits of integrated team action realized (Atwal & Caldwell, 2005; Clarke & Forster 2015). Although this coordination requires time and a commitment to cooperation, substantial benefits result from merging the collective knowledge and specialized skills of diverse rehabilitation professionals. Regarding the case presented herein, the benefit was evident in the incorporation of interdisciplinary activities that encouraged numerous repetitions, served a functional purpose in the client's daily life, and aided her efforts toward further recovery and achievement of long-term goals.

An important aspect of the implemented teamwork was the incorporation of meaningful activities using readily available materials and serving functional purposes within the client's living environment. This represents a potential advantage of telerehabilitation over in-person rehabilitation. Although in-person practitioners may strive to make treatment activities meaningful and personalized, having access to a client's home environment facilitates this process and makes it easy to suggest adaptations and alternative methods of performing difficult tasks (Temkin et al., 1996). For example, instead of practicing stair climbing on an artificial staircase available in a clinic setting, telerehabilitation practitioners use the stairs and railings in a person's dwelling; instead of preparing meals in an adapted kitchen, telerehabilitation practitioners provide meal preparation instruction using tools and equipment available in a client's personal kitchen; and instead of working on reading with generic books or magazines available in a clinic or accessible online, telerehabilitation practitioners use materials a client has and wants to read for leisure or work purposes. The realism and personalization afforded by these types of activities optimize motivation and engagement—two crucial factors given the need for a multitude of repetitions to promote neuroplasticity and maximize recovery.

## STUDY LIMITATIONS

Limitations are inherent when using case examples to examine rehabilitation practices or glean evidence about treatment efficacy. Case examples lack the scientific rigor associated with controlling and manipulating variables for hypothesis testing purposes. Furthermore, specific aspects of the case presented herein add to its uniqueness and lessen the appropriateness of generalizing the results. Specifically, the client was highly knowledgeable about stroke recovery and neuroplasticity because of her profession; she may also have had relatively high cognitive reserve in comparison to other people. As such, the telerehabilitation team did not need to expend effort convincing her of the benefit associated with extensive task repetition; unlike what sometimes occurs with other clients, her full participation in treatment never wavered. Also, the client's relative youth, strong family support, and long-term access to rehabilitation services were advantageous for recovery; not all clients have such advantages or resources. Still, despite these caveats, the client's achievements provide a valid example of post-stroke recovery attained with services delivered by a collaborative team of telerehabilitation specialists working in an interdisciplinary fashion.

## CONCLUSION

Telerehabilitation as an alternative to in-person services has, at least in part, gained popularity because of restrictions imposed by the COVID-19 pandemic. Factors such as client and therapist convenience, cost, and evidence of effectiveness are likely to affect the long-term acceptance of telerehabilitation as a preferred service delivery method. A risk to this acceptance is the potential for professionals to work in isolation rather than as an interdisciplinary team. As such, establishing ways in which professionals can work collaboratively and cooperatively when delivering services via telecommunication technologies is critical. Using an interdisciplinary team approach can serve as a tool to maximize recovery by promoting neuroplasticity through activity repetition and encouraging maintenance and generalization of improved functioning to activities occurring outside scheduled treatment sessions.

## Footnotes

1 Client's identity revealed at her request.

## References

[B1] Agency for Healthcare Research and Quality (2019, March). TeamSTEPPS fundamentals course: Module 3. Communication. https://www.ahrq.gov/teamstepps/instructor/fundamentals/module3/igcommunication.html

[B2] American Physical Therapy Association. (2020). *Impact of COVID-19 on the physical therapy profession: A report from the American Physical Therapy Association*. American Physical Therapy Association. https://www.apta.org/contentassets/15ad5dc898a14d02b8257ab1cdb67f46/impact-of-covid-19-on-physical-therapy-profession.pdf

[B3] Atwal, A., & Caldwell, K. (2005). Do all health and social care professionals interact equally: A study of interactions in multidisciplinary teams in the United Kingdom. *Scandinavian Journal of Caring Sciences*, 19(3), 268–273. 10.1111/j.1471-6712.2005.00338.x 16101855

[B4] Belagaje, S. R. (2017). Stroke rehabilitation. *Continuum: Lifelong Learning in Neurology*, 23(1), 238–253. 10.1212/CON.0000000000000423 28157752

[B5] Buheji, M., & Hassani, A. (2020). Design of lean tele-rehabilitation services for post COVID-19 pandemic. *International Journal of Advanced Research in Engineering and Technology*, 11(11), 1360–1371. 10.34218/IJARET.11.11.2020.123

[B6] Careau, E., Vincent, C., & Noreau, L. (2008). Assessing interprofessional teamwork in a videoconference-based telerehabilitation setting. *Journal of Telemedicine and Telecare*, 14(8), 427–434. 10.1258/jtt.2008.080415 19047453

[B7] Clarke, D. J. (2010). Achieving teamwork in stroke units: The contribution of opportunistic dialogue. *Journal of Interprofessional Care*, 24(3), 285–297. 10.3109/13561820903163645 19995268

[B8] Clarke, D.J., & Forster, A. (2015). Improving post-stroke recovery: The role of the multidisciplinary health care team. *Journal of Multidisciplinary Healthcare*, 8, 433–442. 10.2147/JMDH.S68764 26445548 PMC4590569

[B9] Cramer, S.C., Dodakian, L., Le, V., See, J., Augsburer, R., McKenzie, A., Zhou, R.J., Ghiu, N.L., Heckhausen, J., Cassidy, J.M., Scacchi, W., Smith, M.T., Barrett, A.M., Knutson, J., Edwards, D., Putrino, D., Argawal, K., Ngo, K., Roth, E.J., … Janis, S. (2019). Efficacy of home-based telerehabilitation vs in-clinic therapy for adults after stroke. *JAMA Neurology*, 76(9), 1079–1087. 10.1001/jamaneurol.2019.1604 31233135 PMC6593624

[B10] Heuer, A., Hector, J.R., & Cassell, V. (2019). An update on telehealth in allied health and interprofessional care. *Journal of Allied Health*, 48(2), 140–147. 31167017

[B11] Macoir, J., Sauvageau, V.M., Boissy, P., Tousignant, M., & Tousignant, M. (2017). In-home synchronous telespeech therapy to improve functional communication in chronic poststroke aphasia: Results from a quasi-experimental study. *Telemedicine and e-Health*, 23(8), 630–639. 10.1089/tmj.2016.0235 28112589

[B12] Markle-Reid, M., Orridge, C., Weir, R., Browne, G., Gafni, A., Lewis, M., Walsh, M., Levy, C., Daub, S., Brien, H., Roberts, J. & Thabane, L. (2011). Interprofessional stroke rehabilitation for stroke survivors using home care. *Canadian Journal of Neurological Sciences*, 38(2), 317–334. 10.1017/S0317167100011537 21320840

[B13] Meltzer, J.A., Baird, A.J., Steele, R.D., & Harvey, S. J. (2017). Computer-based treatment of poststroke language disorders: A non-inferiority study of telerehabilitation compared to in-person service delivery. *Aphasiology*, 32(3), 290–311. 10.1080/02687038.2017.1355440

[B14] Miller, K., Chumbler, N.R., Carlson, K., & Daggett, V. (2014). Tele-rehabilitation to promote exercise in Veterans post-stroke: An observational pilot study. *International Journal of Physical Medicine and Rehabilitation*, 2(3), 1–5. 10.4172/2329-9096.1000200

[B15] Moradi, V., Babaee, T., Esfandiari, E., Lim, S.B., & Kordi, R. (2021). Telework and telerehabilitation programs for workers with a stroke during the COVID-19 pandemic: A commentary. *Work*, 68, 77–80. 10.3233/WOR-203356 33427710

[B16] Prabawa, I.M.Y., Silakarma, D., & Widnyana, M. (2021). Telerehabilitation as a physical therapy solution for the post-stroke patient in COVID-19 pandemic situations: A review. *Intisari Sains Medis*, 12(1), 1–5. 10.15562/ism.v12i1.873

[B17] Stein, J., Visco, C.J., & Barbuto, S. (2020). Rehabilitation medicine response to the COVID-19 pandemic. Advance online publication. *American Journal of Physical Medicine & Rehabilitation*. 10.1097/PHM.0000000000001470 PMC7268871 32433243

[B18] Stroke Unit Trialists' Collaboration. (2013). Organised inpatient (stroke unit) care for stroke. *Cochrane Database of Systematic Reviews*. 10.1002/14651858.CD000197.pub3 PMC6474318 24026639

[B19] Tchero, H., Tabue-Teguo, M., Lannuzel, A., & Rusch, E. (2018). Telerehabilitation for stroke survivors: Systematic review and meta-analysis. *Journal of Medical Internet Research*, 20(10), e10867. 10.2196/10867 30368437 PMC6250558

[B20] Temkin, A.J., Ulicny, G.R., & Versarovich, S. H. (1996). Telerehab: A perspective of the way technology is going to change the future of patient treatment. *Rehab Management*, 9(2), 28–30. 10166562

[B21] Tenforde, A.S., Borgstrom, H., Polich, G., Steere, H., Davis, I.S., Cotton, K., O'Donnell, M., & Silver, J. K. (2020). Outpatient physical, occupational, and speech therapy synchronous telemedicine: A survey study of patient satisfaction with virtual visits during the COVID-19 pandemic. *American Journal of Physical Medicine & Rehabilitation*. Advance online publication. 10.1097/PHM.0000000000001571 PMC7526401 32804713

[B22] Yagura, H., Miyai, I., Seike, Y., Suzuki, T., & Yanagihara, T. (2003). Benefit of inpatient multidisciplinary rehabilitation up to 1 year after stroke. *Archives of Physical Medicine and Rehabilitation*, 84(11), 1687–1691. 10.1053/S0003-9993(03)00286-7 14639571

[B23] Yagura, H., Miyai, I., Suzuki, T., & Yanagihara, T. (2005). Patients with severe stroke benefit most by interdisciplinary rehabilitation team approach. *Cerebrovascular Diseases*, 20(4), 258–263. 10.1159/000087708 16123546

